# Understanding practitioner perspectives of youth healthcare using thematic and lexical analyses

**DOI:** 10.1111/hex.12950

**Published:** 2019-08-13

**Authors:** Ann Dadich, Carmen Jarrett

**Affiliations:** ^1^ School of Business Western Sydney University Parramatta NSW Australia; ^2^ NSW Kids and Families NSW Ministry of Health North Sydney NSW Australia

**Keywords:** adolescent health, attitudes, health knowledge, management service organizations, methods, practice, qualitative research

## Abstract

**Background:**

Youth health issues represent a “wicked problem” – they are complex and multifaceted. Furthermore, they are likely to require novel approaches to understand their complexity and develop novel solutions.

**Objective:**

Given the importance of youth healthcare, and the need for novel approaches, the aim of this article was to demonstrate the innovative use of two research methods – thematic and lexical analyses – to better understand practitioner perspectives of youth healthcare. It clarifies the factors that shape practitioner ability to support young people and opportunities to improve practice.

**Design and Setting:**

Focus groups and interviews were conducted with 37 youth health practitioners. They represented government and non‐government services; hospital and community services; and metropolitan and regional services.

**Results:**

Thematic analysis highlighted the complexity of participants' work and the judgements made as they negotiated relationships with young people and ancillary services. Lexical analysis revealed two hitherto neglected dimensions of the complexity of youth healthcare – uncertainty and corporeality.

**Discussion:**

In addition to affirming the complexities of youth healthcare, this study revealed how practitioners (can) negotiate these complexities. These findings were only possible because of the innovative use of the two research methods.

**Conclusions:**

This study has important theoretical, methodological and practical implications. Theoretically, it is the first to view the complexities of youth healthcare through the wicked problem lens. Methodologically, it highlights the complementary value of thematic and lexical analyses. Practically, it reinforces the importance of policy support and professional development to enable practitioners to grasp the complexities of their work.

## 1 INTRODUCTION

Youth (mental) health issues represent a “wicked problem”.^1^ Shaped by nature, socio‐cultural‐economic context, the availability of appropriate services and politics, they are often imprecise and related to moral, political and professional issues. Wicked problems “*are difficult to clearly define*…* multi‐causal*… *often lead to unforeseen consequences*… [un]*stable*… *usually have no clear solution*… *are socially complex*… and… *hardly ever sit conveniently within the responsibility of any one organisation*”.^2^ Accordingly, wicked problems require novel approaches that accommodate multiple perspectives and involve interaction and iteration.^3^

Enticed by wicked problem literature, this article demonstrates the innovative use of two research methods – thematic and lexical analyses – to clarify practitioner perspectives of youth healthcare. It examines the factors that shape practitioner ability to support young people and identifies opportunities to improve practice. By presenting findings from both methods, their complementarity becomes apparent, because together, thematic and lexical analyses magnify nuances in youth healthcare and reveal prospects that might otherwise be missed.

### 1.1 Youth health

Adolescence is a time of rapid change, which requires specific attention from health services. Although young people – 10‐24 years of age – are often healthy, many face significant health threats. Furthermore, the social context in which young people mature is complex.^4^, ^5^ Thus, youth health(care) can differ from adult health(care). Notwithstanding shared experiences between young people and adults – including the rise of chronic health issues and the fragmented ways health services typically address these^6^, ^7^ – young people can develop lifestyle behaviours that shape adult life. Although many are digitally literate, many have limited health literacy, partly because of stigma. Furthermore, traversing fragmented health services can be more difficult when transiting from child to adult services.^8^, ^9^, ^10^, ^11^, ^12^

Youth healthcare can be a challenging area to work, partly because of limited support.^13^, ^14^, ^15^, ^16^ A systematic review suggested youth access to sexual and reproductive health services is hindered by service accessibility; privacy and confidentiality; and staff characteristics and competencies.^17^ Another concluded that youth engagement with health systems is influenced by “the ability to recognize and understand health issues; service knowledge and attitudes toward help‐seeking; structural barriers; professionals' knowledge, skills, attitudes; service environments and structures; ability to navigate the health system; youth participation; and technology opportunities”.^18^ Additionally, many social determinants of youth health lie beyond the remit of most social services. These challenges collectively suggest many young people are likely to experience health issues that have the features of a wicked problem.

### 1.2 Wicked problems

Rittel and Webber^1^ noted that interacting open systems are not discrete and readily measurable, but involve many, changing and potentially competing elements. They are emergent and ill‐defined. Furthermore, Rittel and Webber appreciated the limits of conventional approaches to address these problems. Contemporary problematic social situations were thus coined as “wicked” because they are “tricky”.^1^ Wicked problems involve many stakeholders who often disagree; there is no obvious solution to facilitate behavioural change. Furthermore, tackling the problem often gives rise to new challenges.^19^

The value of the “wicked problem” is not merely its conception, but the suggested solution. Solutions “lie well beyond the traditional domain of any one jurisdiction or organisational entity, and beyond *business‐as‐usual*”.^20^ Solutions require collaborative and engaged inquiry by embracing alternative perspectives; developing visual representations to view the phenomenon in different ways; examining relationships between relevant elements and discrete alternatives; and focusing on possibility rather than probability.

Heeding the call to widen scholarly blinkers, health researchers have found value in the wicked problem lens.^21^, ^22^, ^23^ Health research suggests two points. First, the wicked problem lens can clarify the complexities that pervade healthcare. Second, it is yet to be used to view the complexities of youth healthcare – this article addresses this void.

Given the importance youth healthcare, and the value of different research methods,^24^ this article uses thematic and lexical analyses to develop a different understanding of youth healthcare. These methods offer alternative perspectives; present visual representations of the phenomenon; consider relationships between relevant elements and discrete alternatives; and unveil opportunities to bolster youth healthcare. The use of thematic and lexical analyses makes sound theoretical and methodological sense. This is because they can bolster research capacity to properly focus the problem they are studying.

## 2 METHOD

Ten focus groups were facilitated with 37 practitioners who supported young people in New South Wales, Australia. The purpose of these groups was to inform a clinical resource (see Table 1). Participants purposely included representation from government and non‐government services; hospital and community services; and metropolitan and regional services. Participants were affiliated with services that addressed a health topic, or specifically supported young people. To ensure representation from the disability sector, participants were also sourced from a school that supported young people with special learning needs.

**Table 1:** Discussion schedule

Topic	Example questions
1. Experiences with and perceptions of youth healthcare	How do you typically engage with young people who access your service and support them?
2. Factors that help and hinder youth healthcare	What enables you to fulfil your role?
3. Ways to translate information on evidence‐based practices into healthcare	How have you sourced and used information to guide your practice?

Focus groups were used because they can elicit and cross‐pollinate opinions, enabling participants to build on the ideas of fellow participants^25^; focus group transcriptions are appropriate for thematic and lexical analysis.^26^, ^27^ The focus groups were facilitated between April and June 2012 (inclusive) and transpired for approximately an hour. Guided by relevant literature,^28^, ^29^, ^30^ the facilitator posed queries; provoked discussion with prompts; moderated respectful dialogue; and ensured all participants had opportunity to contribute. Each group involved between two and eight participants, except when two participants were interviewed (due to limited availability). Although these interviews might have limited participant capacity to discuss and debate disparate views, it also enabled the interviewees the opportunity to extend discussion on particular points, thereby enriching the data collected.^31^ Discussions were audio‐recorded and transcribed for analysis. As a quality improvement project, this study was endorsed by the local Service Improvement Unit and lodged by this Unit with The Children's Hospital at Westmead's Ethics Committee.

### 2.1 Analysis

Two forms of analysis were conducted to understand the data from different vantage points – thematic and lexical analyses. The thematic phase involved three overlapping processes (see Table 2) to “decrease, negate, or counterbalance the deficiency of a single strategy, thereby increasing the ability to interpret the findings”.^32^

**Table 2:** Description of thematic and lexical analyses

Analysis	Description
1. Thematic	
1.1. Constant comparison analysis^38^	This involved methodically coding the data and constructing themes from the codes^39^; themes were identified in relation to the focus of the study and were compared between participants
1.2. Optimisation of variance^40^	This involved ensuring that descriptions and explanations about the data contained both typical and atypical elements
1.3. Triangulation through researcher and member checks^41^	Triangulation involved two strategies – first, both authors conducted the analysis. Discussion of their constructed themes helped to increase the rigour and trustworthiness of the findings. Second, the researchers' analysis was checked by an Advisory Group comprised of nine practitioners who, as representatives of the youth health sector, hold expertise in this area
2. Lexical	
2.1. Discovery mode	Once transcripts were aggregated, the “discovery” mode was used to “see what concepts were automatically generated by Leximancer without intervention”.^33^ Illustrating the automatically generated relationships within the text, in the first instance, helps to “create learning and understanding”^42^ and identify ways to make sense of these relationships
2.2. Relevancy weightings	Leximancer was used to examine the relative importance of the concepts, as denoted by relevancy weightings. A relevancy weighting denotes “the relative strength of a concept's frequency of occurrence”^43^
2.3. Tagging	The concepts were examined by tagging the text with participant identifiers. Tagging helps to compare the conceptual content of different data.^44^ Hence, to determine whether (and how) the context in which participants worked influenced their contributions to this study, each response was tagged to identify the service the participant was affiliated with. Leximancer was configured to “Learn From Tags” using a “Supervised” “Learning Type”, and discovering only those “Concepts in Each” to illustrate the exclusive disjunctions – this helped to clarify how responses differed, rather than how they were similar

The lexical phase involved conceptual and relational content analysis, aided by Leximancer^33^ – data‐mining software that uses Bayesian reasoning to detect key concepts and reveal their relationships^34^ (see Table 3). Leximancer was used in three steps (see Table 2). Although the use of Leximancer helped to construct thematically and conceptually rich findings, only the most salient are discussed that address the aim of the article.

**Table 3:** Leximancer description and justification

Description
Using algorithms, Leximancer identifies frequently occurring and co‐occurring words and amalgamates these to form and visually map concepts that reflect topics within the text.^45^, ^46^ The components of these concepts are ordered within a thesaurus, comprised of relevant words and weightings to indicate relative importance. Specifically, the maps convey: “the main concepts in the text and their relative importance; the strengths of links between concepts (how often they co‐occur); and similarities in contexts where links occur”.^47^ Concepts represent “collections of words that generally travel together throughout the text”.^48^ Within the map, connections between concepts that are most probable are represented by a spanning tree of grey lines or branches. Clusters of concepts within a map – known as themes – suggest contextual similarity. For clarity, themes are colour‐coded to signify those that are (and are not) important – “This means that the ‘hottest’ or most important theme appears in red, and the next hottest in orange, and so on according to the colour wheel”^48^
Justification
1. Leximancer can offer a “helicopter” view of a substantial body of qualitative data, illustratively portraying relationships and patterns between representative themes and concepts^49^
2. As a form of computer‐assisted qualitative data analysis software (CAQDAS), Leximancer offers a systematic, logical and an efficient method to text‐mine^50^
3. Unlike thematic analysis,^51^, ^52^, ^53^ Leximancer can help to reveal and make sense of different findings^54^ – given its capacity to offer an “unsupervised” view of the data,^55^ it can facilitate “broader opportunities for interrogating the text”^27^ by grounding the analysis in the participants' voice. As such, Leximancer can direct researcher attention to the unexpected (as well as what might be expected)^56^

**Figure 1:**
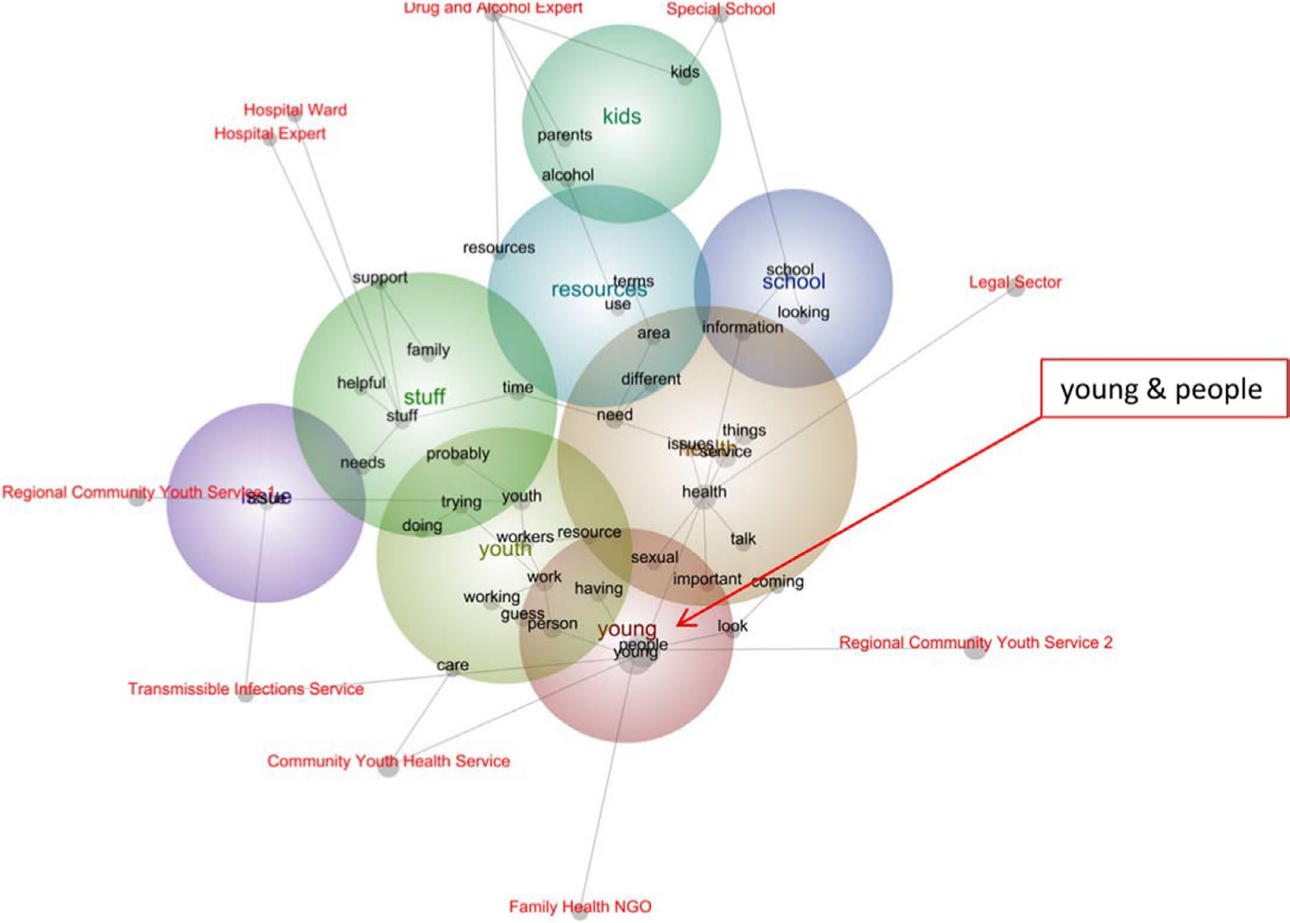
Concept map

Figure 1 depicts relationships between concepts identified by participants from different organisations. Tags indicate participants' organisations. The relative size of the grey points suggests the concepts, “health”, “young” and “people”, were most frequently reported. Furthermore, the latter two are close to each other within the concept map, indicating their relevance. Although the prevalence and location of all three concepts might be expected (given the study focus), surprisingly, they are not equidistant between all ten tags, as explicated in the results.

Relevancy weightings verify the relative strength of relationship between organisations and concepts (see Table 4). For example, the “Transmissible Infections Service” weightings associated with “young” and “people” were 29% and 29%, respectively, compared to the “Hospital Expert” for who the weightings for these concepts were 4% and 4%, respectively. Between these extremes were “Regional‐2” and “Regional‐1” (20% and 20%, and 13% and 12%, respectively).

**Table 4:** Participants' key concepts

Descriptor	Young		People		Health		Service	
	Count^a^	Likelihood (%)^b^	Count^a^	Likelihood (%)^b^	Count^a^	Likelihood (%)^b^	Count^a^	Likelihood (%)^b^
Community Youth Health Service	66	25	63	24	30	11	28	10
Drug and Alcohol Expert	27	9	26	9	14	5	19	6
Family Health NGO	34	25	33	24	15	11	9	7
Hospital Expert	4	4	4	4	5	5	4	4
Hospital Ward	3	4	4	6	2	3	1	1
Legal Sector	56	22	54	21	36	14	12	5
Regional Community Youth Service 1	31	13	29	12	14	6	12	5
Regional Community Youth Service 2	78	20	79	20	51	13	53	13
Special School	10	5	9	5	25	13	22	12
Transmissible Infections Service	35	29	35	29	8	7	7	6

a “*Count* represents the number of times a concept appears in the entire corpus”.^54^

b *Likelihood* denotes the probability that a descriptor is associated with a particular concept – for example, the concept of “young” is most likely to be associated with the contributions of the “Transmissible Infections Service” (29%).

## 3 RESULTS

### 3.1 Thematic analysis

Participants described the challenges of working with young people as they attempted to engage with them. Young people often required age‐appropriate information to support healthy lifestyle choices. Yet meeting information needs was not always straightforward because the material seldom addressed young people's priorities. They often wanted to know how they fared relative to their peers:

Young people are preoccupied with being normal and what's normal, but… there is no normal. (Family Health Non‐Government Organisation – hereafter, NGO)

Young people's needs varied, from the simple – like the need to maintain personal hygiene – to the complex – like the need to recover from trauma. Furthermore, young people often presented with both simple and complex needs. Given such variety, some participants found it difficult to isolate the needs of a young client:

A whole… range of things… For some young people, it's about… a roof over [their] head… money and clothing… It's [also about]… breaking the pattern of distrust. (Regional Community Youth Service 1 – hereafter, Regional‐1)

According to some participants, isolating these needs took time, as young people were said to have limited access to health services. Notwithstanding geographical and financial barriers, participants suggested that young people find the foreign world of “health services” confronting:

The health system is very alienating… if… they have to go and sit in a waiting room… they'll actually avoid it at all costs even when they really need to [be there]. (Regional‐1)

Participants described young clients who preferred to discuss an outstanding debt or accommodation issues, rather than their mental health or substance use issue. This mismatch was particularly apparent when the young person experienced stigma. For instance, young people with a substance use issue or who had been convicted of a crime were often reluctant to discuss their situation with practitioners. Participants attributed this to a reluctance to engage with practitioners. Conversely, those who worked with young people convicted of a crime noted that some practitioners are reluctant to engage with these young people:

We get people pulling a face… [like they're saying], ‘We don't want your clients… we don't like young… criminal[s]’. (Legal Sector)

To address young clients' needs, participants described how they negotiated the relationship. This required time, thought and energy to establish rapport, define its parameters and adapt the terms of engagement as the young person's needs changed. Participants noted how they conveyed and operationalised confidentiality protocols. They also described how they reached out to young clients, particularly when they did not present for an appointment, and how they modelled mutual respect. Each act of care solidified the relationship between the young person and the practitioner, and the connection between the young person and the service. According to some participants, it was important the young client recognise value in the service and the support it could provide.

The biggest challenge we have working at a clinical level is engagement with young people and finding meaning in the service that they're receiving. (Community Youth Health Service)

In addition to intangible elements – like practitioner aptitude – the ability to negotiate relationships with young clients required tangible elements, like an appropriate service model. Participants emphasised the importance of flexibility and responsiveness. They were aware a young person's life can change dramatically within a brief period – furthermore, these changes did not always occur during standard business hours. It was therefore important to demonstrate the youth‐friendliness of a service in the way it operated, including its opening hours and outreach services. This was especially important when supporting young people who led a transient lifestyle:

When a homeless young person comes in, we need something then and there because they might not come back… So, taking the service to them. (Regional Community Youth Service 2 – hereafter, Regional‐2)

Once established, the relationship between a young person and a practitioner needed nurturing because it was the platform to promote youth health. Participants were role models to young clients and needed to demonstrate healthy lifestyle choices. This required them to be mindful of their dietary and smoking habits, and language‐use:

I'm just thinking about some of the work in youth health and it was nothing for [some colleagues]… to… share a packet of cigarettes with a young person, primarily to engage them because that was what you did… But… that's not how we operate here. (Community Youth Health Service)

The intangible and tangible elements epitomised an organisational ethos that supported healthy relationships between practitioners and young people. This ethos was youth‐centric – it served to regularly remind practitioners that their aim was to promote youth health. Achieving this aim required self‐awareness and reflective practice. Participants described how they regularly critiqued their practices and the organisational context that supported their work:

What worked for everyone here… is when people are engaged in learning and feeling like they're part of the process… when there's a constant dynamic flow to the way that you work; when you're learning, taking what you learn from the clients, and you're able to find your own answers or look to other people… around you. (Community Youth Health Service)

Self‐awareness and reflective practice were beneficial for individuals and teams. Individually, these practices refined practitioner knowledge and sharpened their skill set. For instance, the practitioners spoke of developing greater “insight” into their roles and responsibilities; how to connect these with their values and ethical standards; and the limits of their working relationship with young people:

Knowing when you've gone as far as you can go, because it's not always clear… Essentially, I think it is something about best practice… It would be very easy to hang on to clients… not seeing an improvement and feeling a responsibility in getting them better. (Community Youth Health Service)

For teams, self‐awareness and reflective practice strengthened a collective commitment to continuous improvement. When this commitment was demonstrated by managers and those who worked directly with clients, an environment was fostered that expected, embraced and managed change:

There [is]… conscious thought on part of the organisation to make [change]… possible… Manager[s]… creat[e]… bedrock principles… [by] wander[ing] around; you know, the basic principle… of… no judgement. (Regional‐1)

Promoting youth health required an ability to negotiate relationships with other services. Given the complex issues some young clients experienced, the participants often liaised with complementary organisations. Collaboration meant sharing information, making inter‐service links and forging pathways for young people. It was difficult for young clients to negotiate the complexity of inter‐service relationships:

As soon as the kids get to about 16, they have to go into the adult services. Before they had one person looking after them and suddenly they are finding that *they* have to know who to go to [somewhere else]. (Special School)

These features of collaboration meant that, at times, interagency relationships were vexed. Participants struggled to gain access for a young client who did not meet the criteria of another service; and facilitate transitioned care with another organisation due to incongruent philosophies, policies, practices or *pro formas*:

The connectiveness… we're really bad at that… For me, the actual client… issues, we're kind of… okay… It's those broader issues of… moving from… other communities to other sites, other hospitals, other centres. They're the biggest issues. (Legal Sector)

Supporting a young person within the service system involves ascertaining when and how to refer young people to appropriate services and negotiating distinct systems. The challenges of negotiating interagency relationships might reflect the greater complexity of working with (and within) several systems. According to some participants, this requires skill, judgement and time. For instance, time is required to maintain interagency relationships and plan client transition to a complementary service, only when the client can address the issue at hand:

[I can only make the referral when] they're ready to address their drug and alcohol. It is very tricky. There's a lot of ethical issues. (Regional‐1)

According to some, interagency relationships are aided by understanding the culture or philosophy of other services. This enabled them to understand how their own service was perceived by other services and recognise disparate priorities or understandings:

Drug and alcohol [services'] philosophy… is really different to ours. They work on harm minimisation; we work under a Mental Health Act. It's not the same thing at all… they're not going to tell somebody who's got a psychosis [to stop] substance abusing. (Community Youth Health Service)

### 3.2 Lexical analysis

The “Transmissible Infections Service”, the “Community Youth Health Service” and the “Family Health NGO” were more likely to refer to “young” and “people”, relative to their counterparts, particularly those at the opposite end of the map, like the “Hospital Expert”.

As suggested by their juxtaposition to “young” and “people” within the concept map, participants from the “Transmissible Infections Service”, the “Community Youth Health Service” and the “Family Health NGO” largely spoke of young people – these included individual clients as well as young people as a large cohort. Conversely, the “Hospital Expert” spoke chiefly of people who worked with young people:

when I first started, I think that's probably the target – these people who need to learn. (Hospital Expert)

Excerpts from the “Transmissible Infections Service”, the “Community Youth Health Service” and the “Family Health NGO” generally referred to “young”, relative to the “Hospital Expert” or the “Hospital Ward” (see Table 4). Given that young people did not represent the “target audience” of the “Transmissible Infections Service”, this weighting represents a curious find. An analysis of the excerpts associated with this concept, however, suggests the “Transmissible Infections Service” spoke of exceptions to the rule – that is, instances when young people were supported by the service, directly by a staff member or indirectly through another service provider:

People under 24… have come… and straight away, they didn't come back… but we could still be a resource for a youth worker. (Transmissible Infections Service)

Conversely, excerpts from the “Community Youth Health Service” and the “Family Health NGO” spoke of greater direct engagement with young people and ways to support engagement. These excerpts collectively highlight young people's needs, as well as service providers' capabilities to adequately fulfil their role:

[Most] of our young people… [experience] financial problems and general health sort of stuff… if a young person approaches you, a health worker's role is assisting their health literacy… there's lots of information coming from inappropriate sources. (Family Health NGO)

Although excerpts from the “Transmissible Infections Service”, the “Community Youth Health Service” and the “Family Health NGO” regarding “young” people implied an identification with youth – as suggested by references to “*our* young people” (Family Health NGO, emphasis added) – those from the “Hospital Expert” and the “Hospital Ward” alluded to boundary setting. Having worked within adult services, one participant suggested that many staff members “are pretty much getting their youth training on the job”. They suggested, although their clinical role might be defined, their non‐clinical role when working with young people was somewhat nebulous:

If my staff were placed in a situation where they would have to follow everything through, while trying to maintain that rapport, it would sometimes be very strenuous on that relationship they've built. (Hospital Ward)

Despite the study focus, the theme – “health” – is not equidistant between all ten participant tags. Instead, it has an increased likelihood of being noted by the “Legal Sector”, relative to the “Hospital Ward” (Table 4). This suggests that health was more apparent in the discourse of the “Legal Sector”. This participant largely spoke of organisational issues that influenced the delivery of healthcare:

When we've got a young person that has a medical issue, we can't just sort of find somebody out in the community to pick him up. Either a GP's not available; [they] straight out say, ‘No, we don't manage your clientele’, or worse than that, ‘Oh well, we’re going to have to charge you extra if we're going to start managing your kids’. (Legal Sector)

These excerpts were juxtaposed to others which spoke of the delivery or receipt of health services, rather than the organisation or management of these services:

I reckon that can be lost on the staff sometimes, that you're dealing with a 12‐year old, now you're not dealing with an 18‐year old, so your manner needs to be different. (Hospital Ward)

Close to “health” is “service”. This concept was largely discussed by “Regional‐2” (13%) and the “Special School” (12%), relatively more than by the other participants, notably the “Hospital Ward” (see Table 4). The former acknowledged issues within the youth health sector, like limited resources, which influenced organisational capacity to meet young people's varied needs:

Specialist autistic services are few and far between… Teachers are time poor… [and] I would expect all your other services are time poor. (Special School)

Yet, many of these issues were underpinned by philosophy. Participants recognised the importance of youth‐friendly environments that were “flexible but… really responsive” (*Regional‐2*). Although this might be part of official rhetoric, it was not always operationalised within services:

We're… a closed silo… You walk into mainstream high‐schools for example, and you have a whole heap of kids in there that are sitting with all sorts of health issues. I can almost guarantee you wouldn't have a single health person in there… and schools don't really know what to do… Making a connection between health and education makes sense… [but] we're operating in two massively different systems… It's a different culture. (Special School)

Despite these issues, “Regional‐2” spoke of strategies to operationalise a youth‐friendly service model. They described a school‐based nurse clinic that accommodated young people's perceived needs and became a gateway to appropriate healthcare. Although unfunded, the clinic operated because of interagency goodwill:

The headmaster… lobbied for it… They're very proactive about ensuring… the young people come to see you… If… someone's… loitering… I've gone, ‘Do you want to come in?’… and the next minute… they're actually talking about certain things. (Regional‐2)

Conversely, the “Hospital Ward” spoke of services when facilitating the transition of young people from hospital to the community. Services were contacted to connect young people with additional support, or arm staff with relevant information.

**Figure 2:**
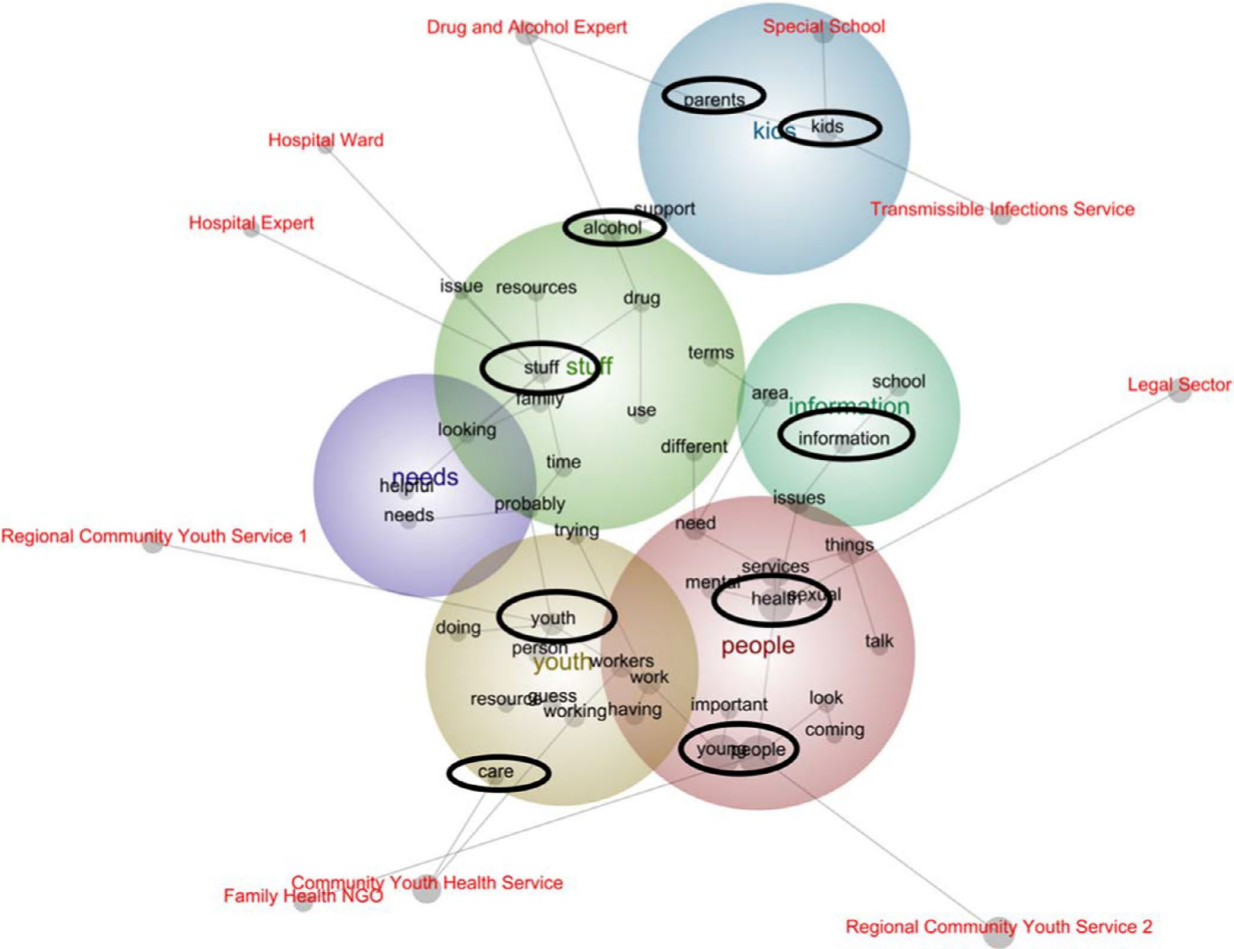
Concept map of exclusive disjunction of themes

Following this examination of key concepts, the next stage involved profiling all concepts according to a nominated set. A map was created to identify specific concepts that were more strongly associated with one participant and not the other, revealing how participant responses differed (see Figure 2). To demonstrate these distinctions, participants are discussed with reference to the concepts that are in closest proximity to their tag and the relevant branches of the spanning tree.

Unlike other participants, the “Legal Sector” mostly discussed the concept “health” (see Table 4). These participants spoke predominantly of health services and, to a lesser extent, youth health issues:

a resource… aimed at a broader audience… would… be really good because [other organisations] just don't seem to get it… We're not talking about individuals, but the organisation as a whole. (Legal Sector)

The “Legal Sector” also spoke about “information”. These participants recognised information as a help and a hindrance. Although there might be considerable documentation about a young person to inform service delivery, it was not always communicated between practitioners – consequently, the young person did not always receive appropriate and/or timely support:

We do have processes in place where we can get that information… but it's not always easy to get it… Good collaborative work doesn't always mean the free‐flow of information. (Legal Sector)

The “Hospital Expert” and “Hospital Ward” spoke mostly about “stuff” (see Table 4). This inclusive concept encompassed references to “preventative health” (Hospital Expert), “assessment”, “their living situation”, “help on homelessness” and “DOCS [Department of Community Services]”, among other matters – all of which were considered “key” (Hospital Ward) to youth work. This concept (“stuff”) speaks to the complexity of youth healthcare. Furthermore, like the limited certainty denoted by “guess” and “probably”, this concept also conveys a degree of ambiguity. Although the participants recognised relationships between youth health, homelessness, and access to government welfare payments, it might not have been possible for them to speak definitively about the strength of these relationships:

we get a lot of those… complex social sort of situations… it would be good to be able to make something tangible… [to guide] other people. (Hospital Ward)

The concept – “stuff” – suggests a preference for tangibility. Like others who spoke of probabilities and “Stuff”, this concept reveals a need for corporeality – a need to grasp the complexity often associated with youth health promotion. This was indicated by participants affiliated with the “Community Youth Health Service”, who spoke of “care” and the associated challenges. These challenges included the tacit dynamics that shape perceptions of and experiences with care – like personal and organisational philosophies. Implicit elements can hinder communication and collaboration with a fellow practitioner, a young person or a family member. Yet, there is something inherently satisfying and affirming when care materialises into an artefact and becomes explicit:

partnership… it's really important [but]… maintaining partnerships is really hard… actually making sure that there's a really clear understanding of each part of the system's model of care and philosophy of care… Concrete, rather than ambiguous [understandings are needed]. (Community Youth Health Service)

While “Regional‐2” and the “Family Health NGO” were in closest proximity to “young” and “people”, “Regional‐1” was closer to “youth”. This suggests, while the former spoke predominately about individuals and their needs, the “Family Health NGO” awarded primacy to youth services and the practitioners, therein:

in a perfect world, a young person would have a GP who was able to provide holistic services and maybe refer… But a lot of the time you see young people, all their needs overlap. (Family Health NGO)

These perspectives present a paradox. It appears that young people's needs were not always addressed or accommodated because of social and political dynamics that influenced the availability of resources and services, perceived or otherwise.

## 4 DISCUSSION

Inspired by literature on wicked problems^19^ and the potential value of approaches that “lie well beyond… *business‐as‐usual*”,^20^ this article presented practitioner perspectives of youth healthcare using thematic and lexical analyses. Together, these methods affirmed the complexities of youth healthcare – notably, the processes of engaging with young people and other services that lie beyond this sector. Furthermore, the findings highlight the capabilities that practitioners require in order to negotiate these complexities. Participants described negotiating their role through interpersonal and intrapersonal judgements and actions, demonstrating the hallmarks of engaged inquiry in pursuit of a collective goal.^20^

This study demonstrates the complementary value of thematic and lexical analyses. While the former affirmed relationship complexity,^35^ the latter highlighted under‐examined dimensions in youth healthcare – uncertainty and corporeality. The lexical analysis indicated that participants experienced uncertainty when engaging with young people and other services. Given the breadth of youth healthcare, they could not be all‐knowing, able to disentangle the messy issues they often confronted. Even highly‐trained practitioners experienced ambiguity, such as when determining if and when to refer a client to a complementary service.

The thematic and lexical analyses illuminate the complexities of youth healthcare. Practitioners required an ability to embrace alternative perspectives as they engage with the uncertainty of youth healthcare – an uncertainty that emerges as they work with young clients and other services that form part of the health system. Furthermore, these perspectives are often imprecise and related to moral, political and professional issues. As hallmarks of a wicked problem, participants alluded to corporeality to concretize the dynamic nature of youth healthcare, transforming the intangible to the tangible and focusing on possibility rather than probability. This penchant for the tangible, revealed by the lexical analysis, might be partly due to the inherent uncertainty of youth healthcare. Participants valued resources that offered practical guidance, while acknowledging that such practicality can be challenging to achieve.

The findings have implications for research, services and policy. For research, this study makes a case for scholarship that tests different strategies to build practitioner skills, especially for continual learning and interagency collaboration; compares distinct service models in youth healthcare; identifies effective ways to articulate health policy; and examines mechanisms to facilitate the translation of policy into practice. Given its demonstrated value,^36^ this might involve practice‐based research – an approach that explicitly recognises and incorporates practitioner perspectives and experiences to redress the conventional privileging of particular forms of evidence, such as that deemed to be scientific or empirical.^37^ This contrasts with research designs that attempt to control contextual nuances that shape organisational practices.

The study also suggests that practitioners require particular skills to engage with young people and other services. Skill development is likely to require more than conventional training. Participants noted the importance of judgement in knowing when and how to refer young people to appropriate services – and they described how experience and intrapersonal resources aided judgement. Thus, professional development was an inter‐ and intrapersonal processes, where learning involved reflection on, and in action, as well as reflecting with others.

For policy, the study suggests that rhetoric alone is insufficient. Despite international recognition of the importance of health service coordination, participants described the challenges of working with other services. This suggests a need to rethink how policy translates into practice. Although professional development might aid this, it is likely to be insufficient. Policies are required that support collaboration, while accommodating the complexity of youth healthcare.

Two limitations warrant mention. First, because participants were self‐selected, there is no claim they constitute a representative sample of practitioners who work with young people. Second, given the research design, it is not possible to isolate the variables – like demographic attributes, training received, professional experience or sector reforms – that contributed to these findings.

Nonetheless, this study is important for theoretical, methodological and practical reasons. Theoretically, it is the first to our knowledge to view the complexities of youth healthcare through the wicked problem lens. This lens helped to widen scholarly blinkers and provoke a consideration of alternative perspectives – perspectives that can be viewed visually, that reveal relationships and that unveil possibility. Methodologically, this study highlights the complementary value of thematic and lexical analyses. Each revealed different understandings of youth healthcare. Practically, this study unveils opportunities to promote youth health. It highlights opportunities to build practitioner skills and refine health policy. These findings are timely given the pressing need for youth healthcare that is both effective and efficient.

## CONFLICT OF INTEREST

A/Prof. Ann Dadich and Dr Carmen Jarrett do not believe there are conflicts of interest to declare.

## Data Availability

The data that support the findings of this study are available on request from the corresponding author. The data are not publicly available due to privacy or ethical restrictions.
